# CRISPR/Cas9-mediated knockout of MLL5 enhances apoptotic effect of cisplatin in HeLa cells *in vitro*

**DOI:** 10.17179/excli2019-1957

**Published:** 2020-01-23

**Authors:** Mohammad Pirouzfar, Farshid Amiri, Mehdi Dianatpour, Mohammad Ali Takhshid

**Affiliations:** 1 Diagnostic Laboratory Sciences and Technology Research Center, Paramedical School, Shiraz University of Medical Sciences, Meshkinfam Street, Shiraz, Iran; 2 Department of Medical Genetics, School of Medicine, Shiraz University of Medical Sciences, Shiraz, Iran; 3 Stem Cell Technology Research Center, Shiraz University of Medical Sciences, Shiraz, Iran

**Keywords:** cervical cancer, CRISPR/Cas9, Mixed lineage leukemia protein, E6 protein, Cisplatin

## Abstract

Mixed lineage leukemia 5 (MLL5) transactivates the expression of E6 and E7 oncogenes in cervical cancer cells. In this study, we utilized CRISPR/Cas9 system with the aim to target HPV-E6 and MLL5 to enhance apoptosis efficiency in HPV-18 positive HeLa cells and to improve chemotherapeutic efficacy of Cisplatin as the most common anticancer drug, used for cervical cancer. sgRNAs against MLL5 and E6 were designed and cloned into PX458 plasmid vector. Real-time PCR was used to determine knockout expression of MLL5 and E6 following, transfection with cloned plasmids. Cell viability and apoptosis were evaluated, using Dimethyl-thiazolyl diphenyl tetrazolium bromide (MTT) assay and Annexin V flow cytometry. ‏Cellular p‎53 level was measured, using enzyme linked immune sorbent assay (ELISA).‏ Real-time PCR indicated the downregulation of E6 and MLL5 in the transfected cells. A significant increase in the accumulation of P53 was observed due to targeting MLL5 and E6 genes. MTT and apoptosis assays showed a significant decrease in cell viability and enhanced apoptosis rate of transfected cells. Combination therapy showed that targeting E6 and MLL5 enhanced apoptotic effect of Cisplatin in MLL5 knockout cells in a synergistic manner. **‏**The results suggest that CRISPR/Cas9 targeting of E6 and MLL5 genes can increase‎ apoptotic effects of Cisplatin and can be considered as a potential combination therapy for the treatment of HPV-‎related cervical cancer.

## Introduction

According to global cancer statistics 2018, cervical cancer (CC) is the fourth cause of malignancies among‎ women, with approximately 570,000 of new cases and 300,000 of deaths per year (Bray et al., 2018[3]). Infection with type 16 and 18 of Human Papillomaviruses (HPVs) is highly associated with the development of CC (Woodman et al., 2007[22]). HPV carcinogenesis is the direct consequence of the integration and expression of E6 and E7 oncogenes in cervical cells (Mighty and Laimins, 2014[18]) as well as disruption of E2 that acts in favor of repressing the expression of the oncogenes (Howley, 1991[10]). E6 and E7 expression contribute to the development of pre-cancerous cervical lesions through deregulation of the expression of P53 and Rb tumor suppressor genes, respectively (Zhen and Li, 2017[24]). The expression of E6 and E7 in CC cells is regulated by the beta isoform of mixed lineage leukemia (β-MLL5) protein that mediates the transcription of E6 and E7 genes *via* forming a complex with the AP-1 transcription factor in Long Coding Region of HPV (Yew et al., 2011[23]). Cheng et al. showed that knock down of MLL5, using siRNA leads to upregulation of P53, p21, and hypophosphorylated Rb in HeLa cells (Cheng et al., 2008[6]). Furthermore, it was revealed that β-MLL5 forms a strong physical complex with P53, inhibiting its ability to bind with chromatin and halts its downstream processes. Yew et al. observed the concurrent downregulation of E6 and E7 in HPV positive HeLa cells and suggesting β-MLL5 as a potential target for treatment of HPV related cervical cancers (Yew et al., 2011[23]). In addition, Nin et al. reported that siRNA-mediated knockdown of β-MLL5 leads to growth inhibition *via* the activation of apoptosis and senescence on tumor growth rate and improves radiosensitization of Cisplatin in HeLa cervical cancer cells *in vitro* and *in vivo* (Nin et al., 2014[19]). All in all, these studies suggest MLL5 as a potential target for treatment of HPV-related cervical cancers.

The current available therapeutic approaches for the treatment of CC patients include radical surgery, radiotherapy, chemotherapy, and chemoradiotherapy (Cadron et al., 2007[4]; Lorusso et al., 2014[17]). However, their efficiency is usually limited by several complications such as loss of fertility and drug resistance. Particularly, CC patients treated with Cisplatin have shown to develop resistance against Cisplatin and other platinum-based drugs, such as Carboplatin (Gore et al., 1989[9]). P53 is well known as the underlying mechanism of increased tolerance against Cisplatin. In fact, activated P53 is a critical element for the appropriate Cisplatin-induced apoptosis (Zhu et al., 2016[27]). Thereby, loss of P53 can impair apoptosis process which leads to tolerance of DNA damage, consequently promoting drug resistance. It was stated that by inducing wild-type P53, the tolerance will resolve and the apoptotic pathways of the cell will be restored (Siddik et al., 1999[21]). 

Clustered regularly interspaced short palindromic repeats (CRISPR)-associated protein 9 (Cas9) is a novel genome editing method with potential therapeutic application in treatment of various disorders (Baliou et al., 2018[1]). It consists of a nuclease moiety (i.e. the Cas9) guided by a short RNA sequence (sgRNA) to the target DNA that exerts a double stranded break (DSB) at the intended site (Jo et al., 2015[14]). CRISPR/Cas9 can be easily programed to disrupt virtually any DNA target by merely exploiting an appropriately designed 20 bp sgRNA (Jinek et al., 2012[13]). Once the DSB is induced, the molecular machinery inside the cell will start to repair the break by a strong but infidel mechanism called non-homologous end joining (NHEJ) process, which ultimately ends in induction of random indel mutations in the DSB site (Bernheim et al., 2017[2]). CRISPR/Cas9 has shown to be effective in knockout of HPV oncogenes with the aim to prevent the tumor growth, and improvement of Cisplatin therapeutic efficiency (Zhen et al., 2016[25]; Hu et al., 2014[12]; Hsu et al., 2018[11]).

In this study, we aimed to disrupt β-MLL5 and E6 genes and examine their effects on viability of HPV18-positive HeLa cells. To this end, we have reprogrammed‎ CRISPR/ Cas9 genome editing system with the purpose of targeting β-MLL5 and E6, and evaluating their effects by measuring the alterations in the P53 protein level, cell viability, cell apoptosis and therapeutic activity of cisplatin.

## Materials and Methods

### Cloning and construction of sgRNA expression PX458 plasmids

**Figure 1:**
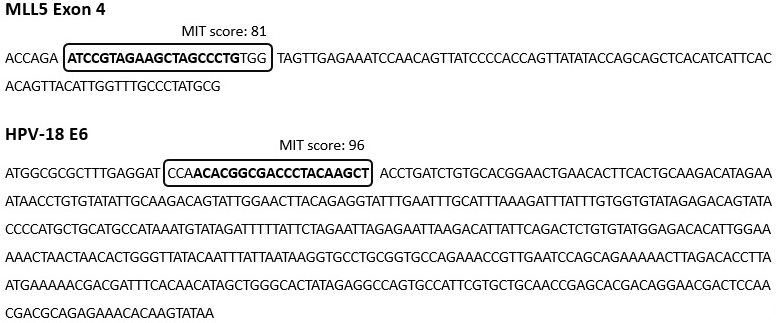
The location of the designed sgRNAs on the coding sequence of the target genes (MLL5 exon 4, and HPV-18 E6). MLL5-sgRNA had the percent peptide of 21 and MIT score of 81, located on the plus strand while the E6-sgRNA was located on minus strand with percent peptide of 5.2 and MIT score of 96.

A series of sgRNAs were designed and analyzed, using sgRNA design tool available at https://crispr.mit.edu/, and DeskGene tool available at https://www.deskgen.com. Two sgRNAs were selected based on their score (the higher efficiency, and the fewer off targets) and their percent peptide. The sgRNAs were synthesized as oligonucleotide DNA sequences and cloned into PX458 plasmid (Addgene, Plasmid #48138) to construct two active CRISPR vectors; One construct targeting MLL5 gene (MLL5-sgRNA: ATCCGTAGAAGCTAGCCCTG) and the other one targeting E6 gene (E6-sgRNA: AGCTTGTAGGGTCGCCGTGT) (Figure 1(Fig. 1)). Cloning was performed according to a protocol described by Ran et al. (2013[20]).

### Cell culture

HeLa and Hek-293 cell lines were purchased by Pasteur Institute, Tehran, Iran. HeLa is an HPV18-positive cell line that consistently expresses E6 and E7 oncogenes as well as MLL5 while Hek-293 is HPV negative, but expresses MLL5. The HeLa and Hek-293 cells were kept and grown in high glucose Dulbecco's Modified Eagle's Medium (DMEM) supplemented with 10 % FBS and 1 % Penicillin/‎Streptomycin (Sigma-Aldrich, USA). 

### Transfection

HeLa and Hek-293 cells in passage three were seeded in six well culture plates. Once the cells reached confluency of 50 %, they were transfected with MLL5-sgRNA, E6-sgRNA or mock PX458 plasmids, using Transfectimine transfection reagent (Dara Zistfan Eram; Shiraz, Iran)‏‎ according to the manufacturer's protocol. Briefly, one tube containing plasmid DNA (9 µg), 200 µL of DMEM, and 10 % FBS, and another tube containing Transfectimine (15 µL), 200 µL of DMEM, and 10 % FBS were prepared. Tubes were kept at room temperature for five min. Afterwards, they were mixed and incubated at room temperature for 20 min. The mixture was poured on the surface of culture media, and the cells were incubated for 8 hours at 37 °C in 5 % CO_2_. At the end of this period, the medium was removed and the cells were carefully washed with PBS, and then cultured in fresh warm complete medium.

### Real-Time PCR

Real time PCR was our method of choice to evaluate the expression of MLL5 and E6 genes in treated target cells. Seventy-two hours after transfection, HeLa cells were subjected to total RNA extraction procedure, using TRYzol RNA Extraction Reagent (Dara Zistfan Eram; Shiraz, Iran). Complementary DNA (cDNA) was synthesized, using PrimeScript 1st strand cDNA Synthesis Kit (Takara, Japan), according to the manufacturer's instructions. qPCR was performed on ABI 7500 Real Time Analyzer (Applied Biosystem, USA), using high-ROX RealQ Plus 2x Master Mix Green (Ampliqon, Denmark) and specific primers for MLL5, E6, and Beta-2-‎microglobulin (B2M) genes (Table 1(Tab. 1)). The experiments were performed independently in a quadruplicate manner.

### Measurement of P53 

The P53 content of HeLa cells was measured, using Human P53 ELISA Kit (Diaclone, France. Cat#: 850.630.048). Seventy-two hours' post-transfection, the transfected cells were lysed and their protein concentration was quantified using Coomassie Brilliant Blue G-250 Dye (Thermo Scientific, USA). One hundred µl of each sample was used for P53 ELISA in a duplicate array. ELISA was performed according to the manufacturer's instructions. P53 concentrations were normalized and reported as percentage of untreated control cells for each sample. 

### MTT assay

Seventy-two hours after transfection, the cells were treated with 28.83 µM (IC_50_) of Cisplatin for 24 hours‏. The treated and control cells (without Cisplatin) were exposed to 10 µL of Dimethyl-thiazolyl diphenyl tetrazolium bromide (MTT; Sigma Aldrich, USA) in concentration of 5 mg/ml‎ and incubated for four hours in 37 °C. Afterwards, 100 μL of Dimethyl sulfoxide (DMSO; Sigma Aldrich, USA) was added to each well and the plates were left in dark for 10 min. Finally, the absorbance of each well was measured in 570 nm wavelength, using an ELISA reader instrument. 

### Flow cytometry analysis of apoptosis

Apoptosis assay was conducted 72 hours after transfection, using PE AnnexinV Apoptosis Detection Kit and FACSCan flow cytometry system (BD bioscience, USA) according to the instructions provided by the manufacturer. A total number of 10,000 events were obtained for each sample within one hour.

### Statistical analysis

All the data were analyzed using IBM SPSS^®^ software version 21.0 (IBM, USA). One-way ANOVA followed by LSD post-hoc comparison test, Mann-Whitney U test, and Kruskal-Wallis test were used for analyzing the parametric and non-parametric data, respectively. *P value* less than 0.05 was considered to be statistically significant.

## Results

### Transfection

**Figure 2:**
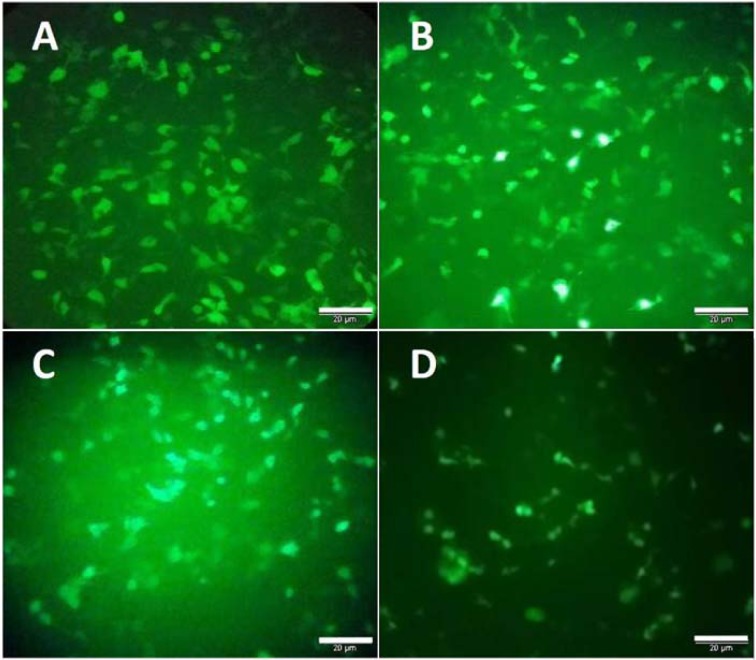
GFP expression was visualized directly by fluorescence microscopy to evaluate the transfection efficiency. A and B represent the Hek-293 cells transfected with PX458 plasmids containing MLL5 and E6 sgRNAs. C and D represent HeLa cells transfected with PX458 plasmids containing MLL5 and E6 sgRNAs, respectively (Scale bars=20µM).

HeLa and Hek-293 cells were transfected with the cloned PX458 plasmid that carries the gene encoding for green fluorescence protein (GFP). The efficiency of transfection was evaluated by counting GFP expressing cells. A high intense fluorescence was observed 24 hours after the transfection, suggesting that the cells were successfully transfected (Figure 2(Fig. 2)). Counting the number of GFP expressing cells in at least five filed of the cultured cells revealed that the transfection efficiency was approximately 70 % and 40 % for the Hek-293 and HeLa cells, respectively. 

### Expression of MLL5 and E6 in HeLa Cells were reduced following transfection with E6 and MLL5 targeting CRISPR/Cas9 constructs 

**Figure 3:**
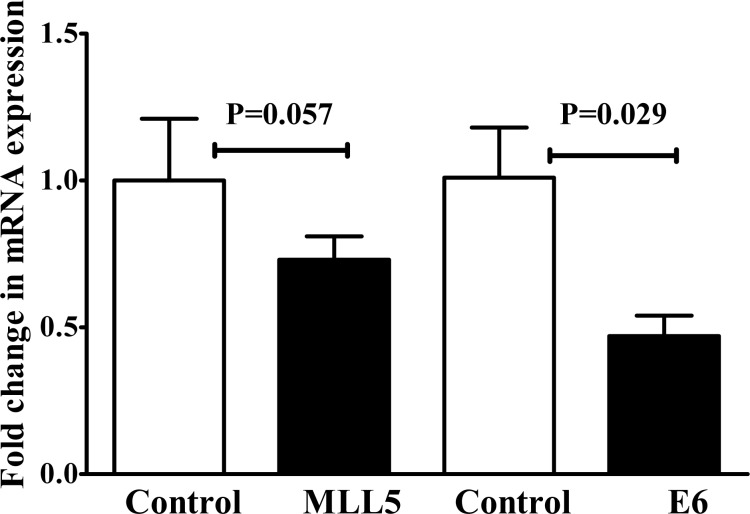
The expression of MLL5 and E6 was reduced 72 hours after HeLa cells were transfected with MLL5- and E6-targeting CRISPR/Cas9 system (MLL5-sgRNA and E6-sgRNA). Represented data are mean ± SD from four independent experiments. Data were analyzed using Mann-Whitney U test and p<0.05 was considered as significant difference between the groups.

Seventy-two hours after transfection, the expression of MLL5 and E6 genes were evaluated, using real time PCR. The results showed a substantial reduction in the level of MLL5 mRNAs in the CRISPR/Cas9 transfected‎ cells compared to untreated control cells. Similarly, a significant reduction (p<0.05) in the level of E6 mRNA expression was observed in the cells transfected‎ with E6 sgRNA compared to untreated control cells (Figure 3(Fig. 3)).

### Elevation of P53 in HeLa cells following transfection with E6 and MLL5 targeting CRISPR/Cas9 constructs 

**Figure 4:**
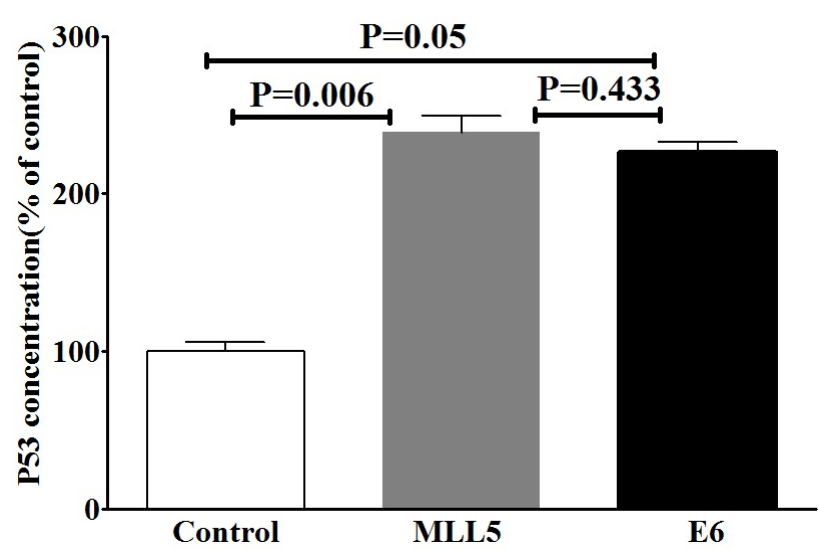
The effects of targeting MLL5 and E6 genes on intracellular accumulation of P53 in HeLa cells. P53 protein was measured using ELISA method and the data was expressed as percentage of the control group. The presented data are mean ± SD. Kruskal-Wallis method was used to analyze the data. P<0.05 was considered as significant difference between groups.

Due to the well-known effect of MLL5 on E6 expression and also the role of E6 on degradation of P53, we evaluated the effects of MLL5 knockdown on intracellular accumulation of P53, using ELISA method. The results of ELISA are summarized in Figure 4(Fig. 4). An increase in P53 protein level was observed following targeting of E6 genes (p=0.05). Similarly, the intracellular accumulation of P53 (‎p=0.006) was increased following targeting the MLL5 gene. No significant difference was observed in P53 levels between the MLL5 and E6 group (p=0.433). 

### The effect of E6 and MLL5 knockout on the viability of HeLa and Hek-293 cells

**Figure 5:**
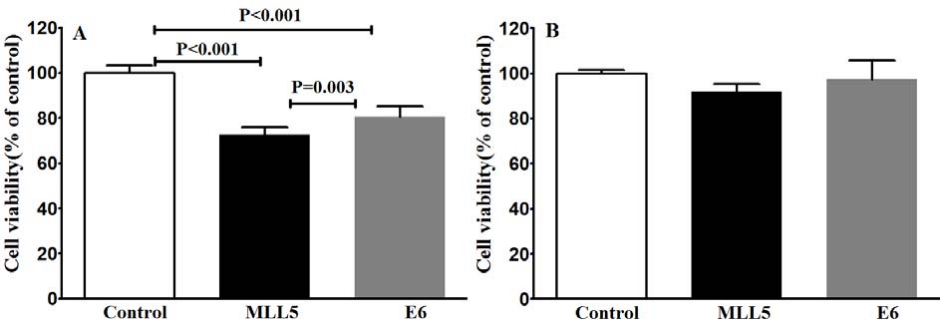
Evaluating viability of HeLa (A) and Hek-293 (B) cells using MTT assay after CRISPR/Cas9-targeting of MLL5 and E6. The presented data are mean± SD of at least four independent experiments. Data were analyzed, using one-way ANOVA followed by LSD post-hoc comparison test. P<0.05 was considered as significant difference between groups.

To examine whether the knockout of E6 and MLL5 would affect the cell viability, MTT assay was carried out four days after transfection. A statistically significant reduction in cell viability was observed in the HeLa cells following disruption of MLL5 and E6 in comparison with the untreated control group while in Hek-293 cells transfection with both MLL5 and E6 sgRNA had no significant effect on cell viability (Figure 5(Fig. 5)). 

### The effect of E6 and MLL5 targeting CRISPR/Cas9 constructs on the efficiency of Cisplatin on HeLa Cells 

**Figure 6:**
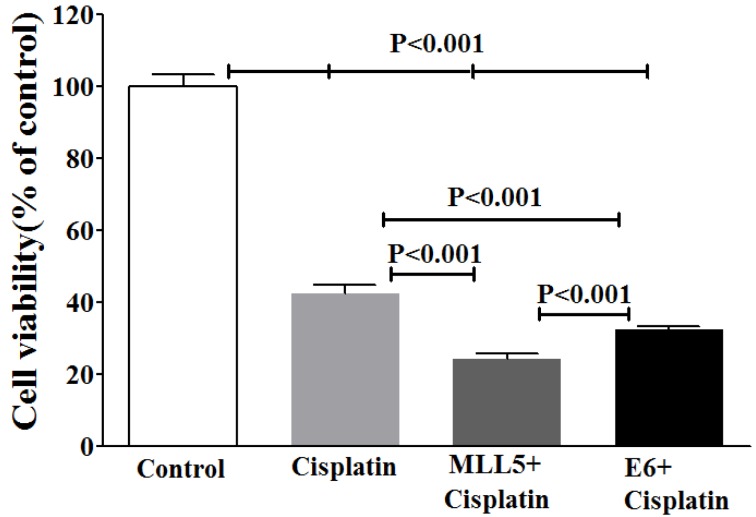
Evaluating viability of HeLa cells using MTT assay after CRISPR/Cas9-targeting of MLL5 and E6 with and without Cisplatin. The? represent data are mean ± SD of at least five independent experiments. Data were analyzed, using one-way ANOVA followed by LSD post-hoc test. P<0.05 was considered as significant difference between groups.

To examine whether the knockout of E6 or MLL5 would increase the Cisplatin efficiency, MTT assay was carried out on day five by treating cells with equal amounts of plasmids for each group, in combination with Cisplatin at its IC_50_ (28.83 µM). Results of the MTT assay exhibited strong synergisms between CRISP/Cas9 targeting of E6 or MLL5 as well as anti-proliferative efficiency of Cisplatin (Figure 6(Fig. 6)).

### Annexin V based apoptosis detection

**Figure 7:**
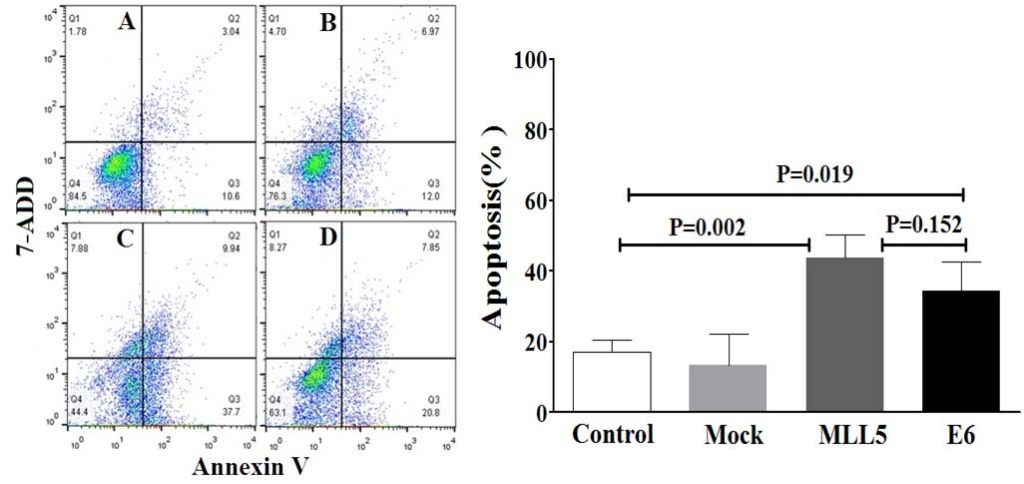
The effect of MLL5-sgRNA and E6-sgRNA on the apoptosis of HeLa cells. Seventy-‎two hours after transfection with MLL5-sgRNA, E6-sgRNA, or mock plasmids the total number of apoptotic cells was quantified, using Annexin V/‎‎7AAD flow cytometry method. Data is expressed as percentage of apoptotic cells in 10000 events per sample. A: Untreated control cells, B: Cells transfected with PX458 Mock plasmid, C: Cells transfected with plasmids harboring MLL5-sgRNA, D: Cells transfected with plasmids harboring E6-sgRNA. In each plot, Q1, Q2, Q3, and Q4 are necrotic (Annexin V-/7AAD+), late apoptotic (Annexin V+/7AAD+), early apoptotic (Annexin V+ and 7-AAD-), and viable (Annexin V- and 7-‎AAD-) cells, respectively. On the graph, presented data are mean ± SD of total apoptotic cells. Data were analyzed using one-way ANOVA followed by LSD post-hoc test for multiple comparison. P > 0.05 was considered as statistical difference.

**Figure 8:**
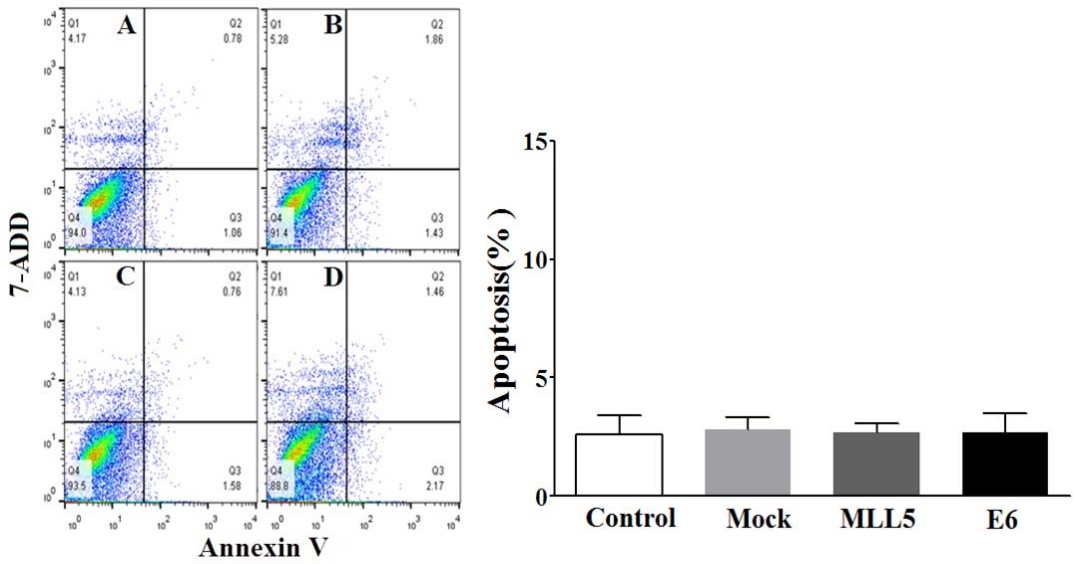
The effect of MLL5-sgRNA and E6-sgRNA on the apoptosis of Hek-293 cells. Seventy-‎two hours after transfection with MLL5-sgRNA, E6-sgRNA, or mock (empty PX458) plasmids the total number of apoptotic cells was quantified, using Annexin V/‎‎7AAD flow cytometry method. Data is expressed as percentage of apoptotic cells in 10000 events per sample. A: Untreated control cells, B: Cells transfected with PX458 mock plasmid, C: Cells transfected with plasmids harboring MLL5-sgRNA, D: Cells transfected with plasmids harboring E6-sgRNA. In each plot, Q1, Q2, Q3, and Q4 are necrotic (Annexin V-/7AAD+), late apoptotic (Annexin V+/7AAD+), early apoptotic (Annexin V+ and 7-AAD-), and viable (Annexin V- and 7-‎AAD-) cells, respectively. On the graph, presented data are mean ± SD of total apoptotic cells. Data were analyzed using one-way ANOVA followed by LSD post-hoc test for multiple comparison. No significant differences were observed between various groups at P > 0.05.

**Figure 9:**
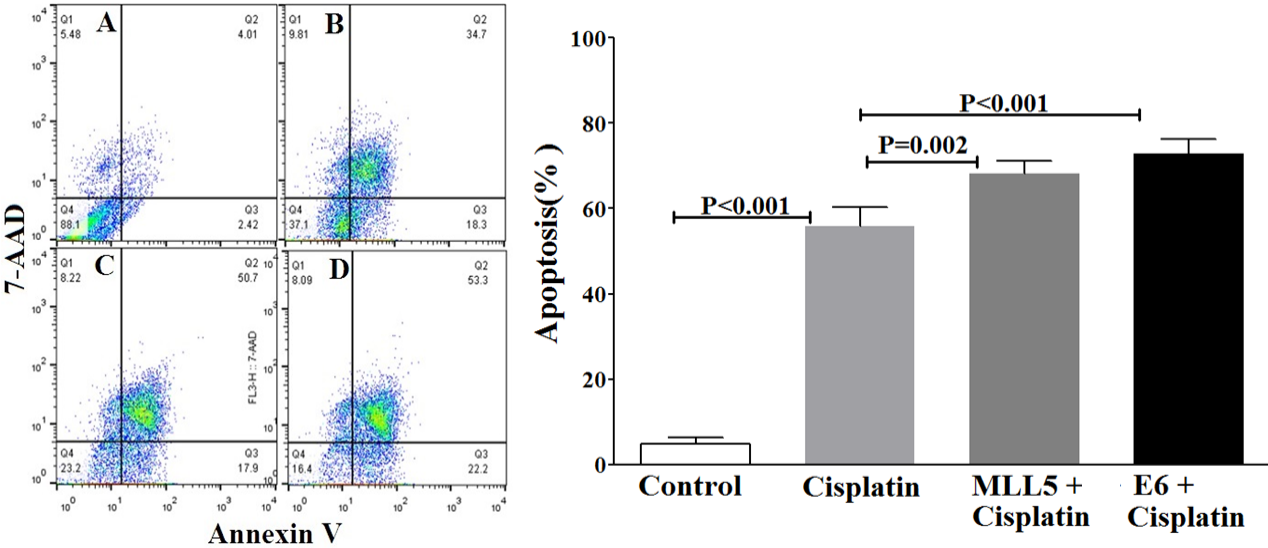
The effect of MLL5-sgRNA and E6-sgRNA alone or in combination with Cisplatin on the apoptosis of HeLa cells. Two days after transfection with MLL5-sgRNA, E6-sgRNA, or mock plasmids, the cells were treated with Cisplatin at its IC_50_. Twenty-four hours later the total number of apoptotic cells was quantified, using Annexin V/‎‎7AAD flow cytometry method. Data is expressed as percentage of apoptotic cells in 10,000 events per sample. A: Untreated control cells, B: Cells transfected with PX458 Mock plasmid, C: Cells transfected with plasmids harboring MLL5-sgRNA, D: Cells transfected with plasmids harboring E6-sgRNA. In each plot, Q1, Q2, Q3, and Q4 are necrotic (Annexin V-/7AAD+), late apoptotic (Annexin V+/ 7AAD+), early apoptotic (Annexin V+ and 7-AAD-), and viable (Annexin V- and 7-‎AAD-) cells, respectively. On the graph, presented data are mean ± SD of total apoptotic cells. Data were analyzed using one-way ANOVA followed by LSD post-hoc test for multiple comparison. P > 0.05 was considered as statistical difference.

The apoptotic status of transfected cells was quantified, using Annexin V/7ADD flow cytometry method. Results indicated significant apoptotic activities of E6 and MLL5 targeting CRISPR/Cas9 constructs in HeLa cells (Figure 7(Fig. 7)) but not in Hek-293 cells (Figure 8(Fig. 8)). Moreover, treatment of HeLa cells with mock plasmid lead to no statistically significant apoptotic effects.‎ In addition, flow cytometry results indicated a significant increase in apoptosis of HeLa cells treated with MLL5-sgRNA or E6-sgRNA combined with Cisplatin in comparison with the cells treated with Cisplatin alone; further suggesting the synergistic effect of CRISPR/Cas9-targeting of MLL5 and E6 on chemotherapeutic efficiency of Cisplatin (Figure 9(Fig. 9)). Additionally, no significant difference was observed between MLL5-sgRNA+Cisplatin and E6-sgRNA+Cisplatin groups.

## Discussion

The recent progresses in the CRISPR/ Cas9 gene editing technology have led to new opportunities in cancer gene therapy (Baliou et al., 2018[1]; Lino et al., 2018[16]). Cancers are heterogeneous complex molecular disorders, resulting by abnormalities in a numerous of genes and signaling pathways. Thus, selecting of appropriate target gene is the most critical step which determines the success of gene therapeutic methodologies including CRISPR/Cas9. The rationale for choosing MLL5 gene in the current study was its regulatory role in proliferation of HeLa cells by binding to P53 and controlling its bioavailability. Moreover, human MLL5 gene is expressed as full-length MLL5, MLL5α, and NKp44L isoforms in many human tissues while it is exclusively expressed as MLL5β in HPV16/18-cervical cancer cells, where it participates in activation of E6/E7 transcription. These characteristics make MLL5 gene an ideal target for the gene therapy of HPV16/18 positive CC. In this regard, some previous studies have evaluated the effects of slicing of MLL5β transcription, using siRNA or inhibition of post translational acylation of MLL5β on viability and apoptosis of CC cells (Yew et al., 2011[23]; Cheng et al., 2008[6], 2011[5]; Nin et al., 2014[19])*. *To the best of our knowledge, the present study is the first to utilize CRISPR/Cas9 technology for targeting MLL5 in combination with Cisplatin, and to evaluate the effects of this knockout on the viability and apoptosis of HeLa CC cells. Our findings revealed a significant increase in P53 level, reduction in cell viability, and increase in apoptosis of HeLa cells following targeting of both MLL5 and E6 genes. Cytotoxicity of Cisplatin was also increased following knockout of MLL5 and E6 genes. These findings suggest that CRISPR/Cas9-mediated knockout of ‏MLL5 and E6 in combination with chemotherapy, might have potential therapeutic benefits in the treatment of HPV16/18 positive cervical cancer.

The first and critical step in the CRISPR/ Cas9 gene editing technology, which determines its specificity, is the design of the CRISPR sgRNA with the highest activity score and the least off-targets. In the present study, we designed and synthesized a sgRNA that targeted exon 4 within the MLL5 gene. This sgRNA can disrupt β-isoform of MLL5 which is specifically expressed in HeLa cells. The results of real time PCR revealed a reduction in MLL5 expression in the HeLa cells, following transfection with the plasmids harboring MLL5-sgRNA. Similar results were obtained for E6 gene in HeLa cells treated with E6-sgRNA plasmid. These findings indicated that knocking out of MLL5 and E6 had efficiently occurred within the targeted regions.

Our data clearly exhibits a significant decrease in viability of HeLa cells but not Hek-293 cells, following knock out of MLL5 and E6, suggesting the specificity of this method in reducing viability of HPV-positive cells which is consistent with the results reported by Nin et al. (2014[19]) and Kennedy et al. (2014[15]). In line with the findings of previous studies (Yew et al., 2011[23]; Nin et al., 2014[19]; Hu et al., 2014[12]), results of flow cytometric apoptosis assay revealed a significant increase in the rate of apoptosis in HPV-18 positive cells following knockout of both E6 and MLL5 genes, a phenomenon not observed in Hek-293 cells, further suggesting the specificity and also the role of apoptosis in the anti-viability effects of our approach. Finally, the results of cell viability and apoptosis assays clearly showed that knockout of both MLL5 and E6 had synergistic effects on the anti-cancer efficiency of Cisplatin, which is in correlation with previous findings by others;* In vitro *and *in vivo* studies conducted by Zhen et al., which showed that HPV16 E6/E7-CRISPR/Cas9 could effectively and specifically increase the cell-sensitivity to Cisplatin (Zhen et al., 2016[25]). Furthermore, Nin et al. (2014[19]) revealed anti-proliferative and proapoptotic effects of siRNA-mediated silencing of MLL5β in HeLa cells both *in vitro* and xenograft tumor models. They also demonstrated that cytotoxic effect of gamma irradiation on viability of HeLa cells to increase, following MLL5β silencing while its overexpression reduced the effect of Cisplatin on radio-sensation of these cells (Nin et al., 2014[19])*.*

Considering the well-known effect of E6 on disruption of P53, and also the potential indirect effect of MLL5 on the bioavailability of P53, which is mediated through increasing E6, we evaluated the effects of E6 and MLL5 knockout on the cellular accumulation of P53 in transfected HeLa cells. The results of P53 ELISA assay revealed a significant increase in cellular P53 accumulation in transfected cells compared to non-transfected control group. A finding that provided a plausible explanation for the reduced cell proliferation and increased apoptosis, and the elevation in sensitivity to Cisplatin as observed in E6 and MLL5-knockout cells. This finding is in agreement with the results of others who reported that cellular level of P53 is elevated following both MLL5 and E6 silencing (Yew et al., 2011[23]; Cheng et al., 2008[6]).

In conclusion, our results further indicated the role of MLL5 in carcinogenesis of HPV positive cervical cancer cells. Moreover, we found that CRISPR/Cas9 mediated disruption of MLL5 has a higher negative impact on the viability of cancer cells in comparison with that of E6. This might be due to the simultaneous contribution of MLL5 to the expression of both E6 and E7; in addition to its role in degradation of P53. For the first time, we revealed that MLL5 knockout has a significant impact on the chemotherapeutic efficiency of Cisplatin in HPV-18 positive cells. All in all, these findings suggest MLL5 as a possible novel target for the treatment of CC.‎ However, this would not result in a desirable action unless an efficient and tissue specific CRISPR expression system is facilitated; a goal being pursued by several research teams with promising results (Eoh and Gu, 2019[8]; Chira et al., 2018[7]; Zhu et al., 2018[26]).

## Notes

Mehdi Dianatpour and Mohammad Ali Takhshid (Diagnostic Laboratory Sciences and Technology Research Center, Paramedical School, Shiraz University of Medical Sciences, Meshkinfam Street, Shiraz, Iran; Tel-Fax: +987132289113, E-mail: takhshidma@sums.ac.ir) equally contributed as corresponding authors.

## Acknowledgement

This manuscript was extracted from the MSc thesis of Mohammad Pirouzfar and was supported by a grant (1396-01-10-14215) from the Vice-Chancellor for Research Affairs of Shiraz University of Medical Sciences Shiraz, Iran. We are also grateful to all staff of Department of Medical Genetics and Diagnostic Laboratory Sciences and Technology Research Center of Shiraz University of Medical Sciences for technical assistance in this work. The authors wish to thank Mr. H. Argasi at the Research Consultation Center (RCC) of Shiraz University of Medical Sciences for his invaluable assistance in editing this manuscript. 

## Conflict of interest

The authors declare that they have no conflict of interest.

## Figures and Tables

**Table 1:**
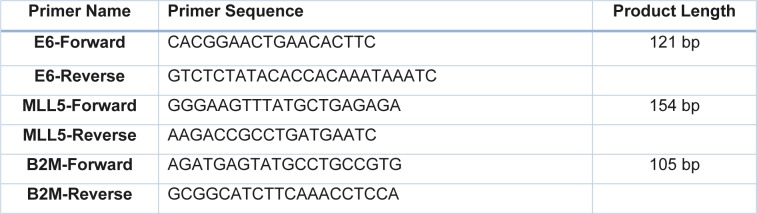
Primers used for quantitative real time PCR
